# Global Proteome Changes in Liver Tissue 6 Weeks after FOLFOX Treatment of Colorectal Cancer Liver Metastases

**DOI:** 10.3390/proteomes4040030

**Published:** 2016-10-14

**Authors:** Jozef Urdzik, Anna Vildhede, Jacek R. Wiśniewski, Frans Duraj, Ulf Haglund, Per Artursson, Agneta Norén

**Affiliations:** 1 Department of Surgical Sciences, Uppsala University, SE-75185 Uppsala, Sweden; frans.duraj@surgsci.uu.se (F.D.); ulf.haglund@akademiska.se (U.H.); agneta.noren@akademiska.se (A.N.); 2 Department of Pharmacy, Uppsala University, SE-75237 Uppsala, Sweden; Anna.Vildhede@pfizer.com (A.V.); per.artursson@farmaci.uu.se (P.A.); 3 Department of Proteomics and Signal Transduction, Max-Planck-Institute of Biochemistry, Martinsried 82152, Germany; jwisniew@biochem.mpg.de

**Keywords:** oxaliplatin-based chemotherapy, protein expression, label-free liquid chromatography mass spectrometry, DNA replication, minichromosome maintenance complex, innate immune response, recovery of liver injury

## Abstract

(1) Oxaliplatin-based chemotherapy for colorectal cancer liver metastasis is associated with sinusoidal injury of liver parenchyma. The effects of oxaliplatin-induced liver injury on the protein level remain unknown. (2) Protein expression in liver tissue was analyzed—from eight patients treated with FOLFOX (combination of fluorouracil, leucovorin, and oxaliplatin) and seven controls—by label-free liquid chromatography mass spectrometry. Recursive feature elimination–support vector machine and Welch *t*-test were used to identify classifying and relevantly changed proteins, respectively. Resulting proteins were analyzed for associations with gene ontology categories and pathways. (3) A total of 5891 proteins were detected. A set of 184 (3.1%) proteins classified the groups with a 20% error rate, but relevant change was observed only in 55 (0.9%) proteins. The classifying proteins were associated with changes in DNA replication (*p* < 0.05) through upregulation of the minichromosome maintenance complex and with the innate immune response (*p* < 0.05). The importance of DNA replication changes was supported by the results of Welch *t*-test (*p* < 0.05). (4) Six weeks after FOLFOX treatment, less than 1% of identified proteins showed changes in expression associated with DNA replication, cell cycle entry, and innate immune response. We hypothesize that the changes remain after recovery from FOLFOX treatment injury.

## 1. Introduction

Preoperative chemotherapy for colorectal liver metastases (CRLM) plays an important role in the multimodal treatment strategy. Liver resection is the only curative treatment and additional preoperative chemotherapy can convert initially non-resectable CRLM to resectable disease [1] or prolong disease-free survival in primary resectable patients [2]. Oxaliplatin-based treatment regimens, such as a fluorouracil, leucovorin, and oxaliplatin combination (FOLFOX), is commonly used as first-line chemotherapy for CRLM. Oxaliplatin-based treatment is, however, associated with sinusoidal injury (SI) in the liver parenchyma, which is reported in 5% [3] to 50% [4,5] of treated patients. Severe SI is clinically associated with increased perioperative bleeding and increased postoperative morbidity [6,7], usually without clinical manifestation of hepatotoxicity during or after therapy [8]. Several clinical studies show that the effect of oxaliplatin-based treatment is reversible and that the liver recovers after chemotherapy cessation [9,10]. Some patients develop SI after only a short period of treatment, while others do not develop SI despite prolonged treatment. This evokes the hypothesis of an individual susceptibility to oxaliplatin-induced injury [11]. The association of SI development with polymorphisms in the nucleotide excision repair genes ERCC2 [12], copper transporter ATP7B [13], and glutathione S-transferase M1 [14] supports the hypothesis.

The exact molecular pathway behind the oxaliplatin-induced liver parenchyma injury remains unclear. Microarray studies attempting to investigate the whole panorama of changes associated with oxaliplatin-based treatment and SI development in humans show an involvement of angiogenesis, cellular adhesion, oxidative stress, and extracellular matrix components [11,15] together with activation of acute phase response, coagulation system, hepatic fibrosis, and hypoxic factors [15]. The role of the mentioned processes is supported by the findings of several studies focusing on particular pathways: angiogenesis [16], oxidative stress [16,17,18,19,20], extracellular matrix remodeling [21,22], and prothrombotic changes [11,23]. However, these changes can also be explained by the presence of CRLM itself [24]. Acute hepatocyte injury caused by the exposure of cultivated hepatocytes to cisplatin (platinum compound similar to oxaliplatin) showed a large proportion (29%) of changes in the proteome [25].

The present study attempts to evaluate the effects of FOLFOX treatment on normal human liver tissue. Changes in protein expression were quantified using label-free liquid chromatography–tandem mass spectrometry (LC–MS/MS) and were investigated for associations with biological processes and pathways.

## 2. Results

### 2.1. Clinical Data

During the study period, 47 patients resected for CRLM donated liver tissue samples to the biobank. Seven patients had no chemotherapy prior to liver surgery and represented a control group. Thirteen patients received preoperative FOLFOX treatment without any biologic agents, and eight of them were randomly selected for the treated group. Patients in the treated group received a median of 5 cycles (interquartile range (IQR) 5–6) of FOLFOX with a median interval of 6 (IQR 5–8) weeks between the last treatment and surgery. Patients were on average 59 years old (IQR 58–69), with a majority of males, 73% (11/15), and had an average body mass index (BMI) of 26 kg/m^2^ (IQR 24–30). There was no difference in clinical characteristics between the groups; for details see Supplementary Material Table S1.

### 2.2. Proteome Description

**Figure 1:**
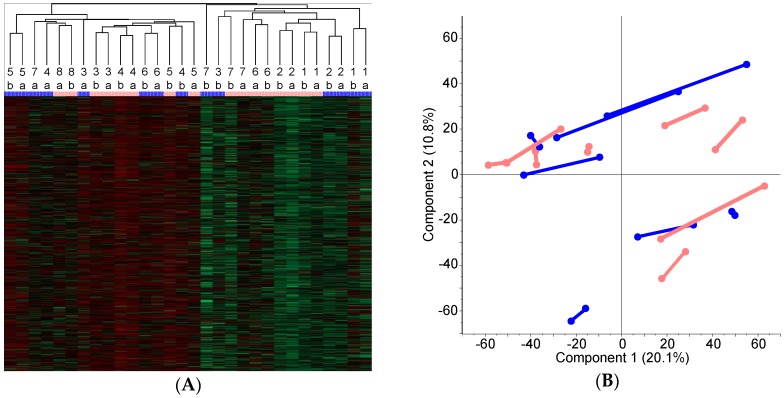
Proteome data description: (**A**) Unsupervised hierarchical clustering using average Euclidean distance, pink color for FOLFOX-treated patients, blue color for controls. Patient number and technical replicates marked with a or b are provided. Treated and control patients were mixed together in final two clusters. (**B**) Principal component analysis (PCA), FOLFOX group in pink and controls in blue, pairs of technical repeats are joined with interconnecting lines. No obvious separation was detected by PCA.

LC–MS/MS analysis allowed identification of 58,757 unique peptides matching to 6689 unique proteins in the liver samples, and 5891 unique proteins that were identified in >50% of the samples were subjected to statistical analysis. Unsupervised hierarchical clustering according to average Euclidean distance (Figure 1A) showed that 10 of 15 (67%) technical pairs were grouped together at the first order of clustering. The treated patients were, however, mixed with controls in around 50% of the final two clusters, as shown in Figure 1A. Principal component analysis (PCA) showed a similar pattern of compact dataset with no obvious discriminating component between the treated and nontreated group. A scatter plot of component **1** (explaining 20.1% of data distribution) versus component **2** (10.8%) revealed an obvious shift between the technical replicates in both groups, mainly in the direction of component **1** (Figure 1B). After subtraction of component **1**, no remaining intraindividual shift was observed. The intraindividual variability was less than the interindividual variability based on the PCA scatter plot. FOLFOX treatment did not induce changes in protein patterns that were detectable by unsupervised hierarchical clustering or PCA.

### 2.3. Classification of the FOLFOX-Treated and Control Group on the Basis of Protein Expression

**Figure 2:**
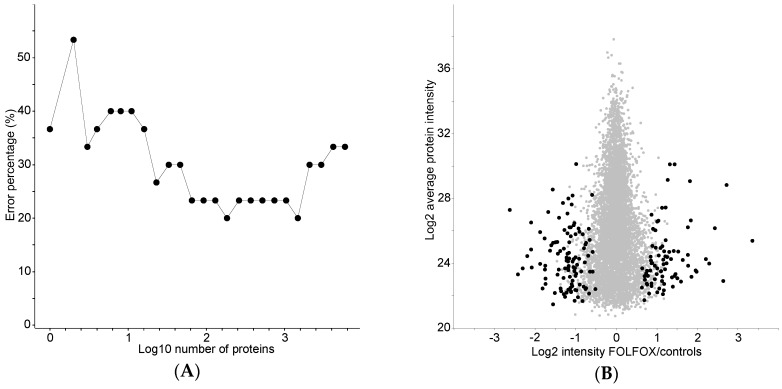
Classification feature optimization method utilizing recursive feature elimination–support vector machine. All identified proteins were ranked according to their classification ability, used in model learning, and cross-validated by leave-one-out method. Logarithm of number of the proteins used in model was plotted against classification error rate (**A**) and average protein change was plotted against average protein intensity (**B**), with the 184 best classifying proteins giving a classification error rate of 20% marked in black and the rest of proteins in grey.

Classifying proteins between the treated group and controls were identified using recursive feature elimination–support vector machine (RFE–SVM) feature optimization algorithm with an attempt to reach high power of enrichment analysis. The smallest number of the proteins providing the minimal classification error rate of 20% was 184 (Figure 2). These 184 proteins are listed in rank order in Supplementary Material Table S2.

### 2.4. Proteome Differences between FOLFOX-Treated and Control Group

**Figure 3:**
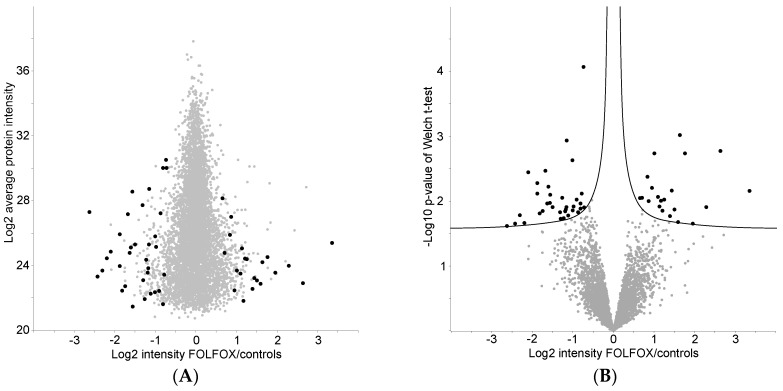
Welch *t*-test relevantly changed proteins, *p*-value < 0.05, false discovery ratio < 0.05, s_0_ = 0.05. (**A**) Logarithm of FOLFOX-treated patients and controls intensities ratio is plotted against average signal intensity of protein and (**B**) against Welch *t*-test *p*-value, with relevantly changed proteins in black and the rest of proteins in grey. Proteins under s_0_ curves but over log-transformed *p*-value threshold were statistically significant, but their biologic effect was judged as marginal.

Welch *t*-test identified 46 (0.8% of all identified) proteins that showed a significant difference in abundance between the treated and nontreated group (*p*-value < 0.05, false discovery ration (FDR) < 0.05). Most of the identified proteins were found in the lower half of the LC–MS/MS dynamic range (i.e., expressed in low abundance, Figure 3A). After manual optimization of s_0_ parameter to 0.05, 55 (0.9%) proteins were recognized as statistically significant and biologically relevantly changed (Figure 3B). Twenty-one proteins were upregulated in the treated group vs controls, with a median fold change of +2.4 (IQR 2.0–3.2) while 34 were downregulated with a median fold change of −2.4 (IQR −3.3 to −2.0). For the complete list of changed proteins, see Table 1.

### 2.5. Protein Ontology and Pathway Analysis

**Figure 4:**
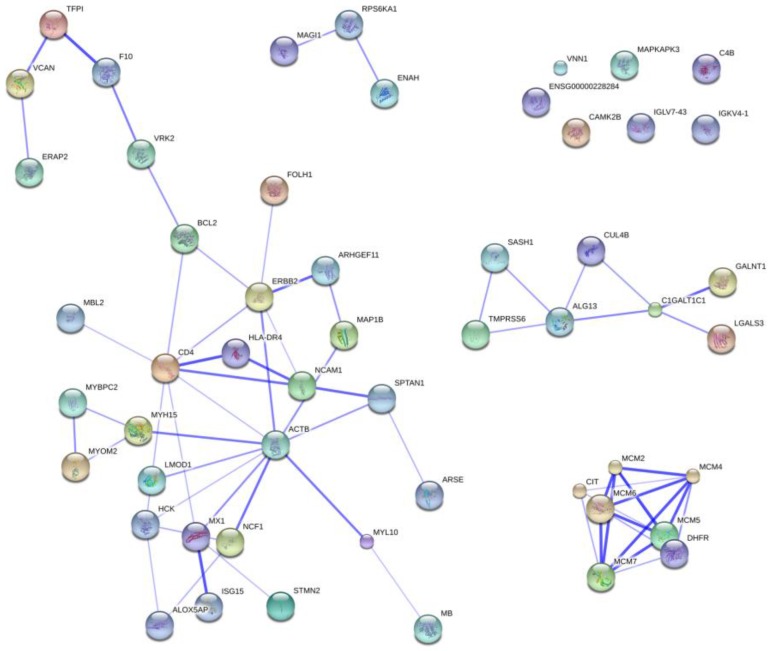
Protein interaction map. Proteins intersecting with ontology or pathway categories (Table 1) are visualized together with proteins interacting with them from classifying model identified list (Supplementary Material Table S2). Thicker lines represent stronger associations.

The proteins included in the RFE–SVM classifying model showed significant association with the DNA replication pathway (FDR corr. *p*-value 0.021). A higher abundance of the minichromosome maintenance (MCM) complex proteins—MCM2, MCM4, and MCM7—was observed in the treated group (FDR corr. *p*-value < 0.001), as seen in Table 2. This complex is involved in the process of DNA unwinding during replication. Moreover, the innate immune response process was also associated with proteins in the RFE-SVM model (FDR corrected *p*-value = 0.029). Enrichment analysis of the proteins with relevantly different abundance in the treated group compared to controls verified the role of the abovementioned DNA replication pathway and process of DNA unwinding. Interaction enrichment analysis in STRING showed significantly more observed protein-protein interactions than expected by chance in the significantly different and RFE-SVM protein groups (*p* < 0.001). The most confident interactions were observed between the MCM complex proteins, but proteins associated with the innate immune response were interacting with the whole network of identified proteins (Figure 4).

## 3. Discussion

This study documented liver parenchyma proteome changes, 6 weeks after FOLFOX treatment, in about 1% of identified proteins. The proteome changes were associated with upregulation of the MCM complex, which—by the process of DNA unwinding—increases DNA replication and indicates cell cycle entry. Observed changes after FOLFOX treatment remain in non-tumorous liver tissue at the time of liver surgery. To our knowledge, this is the first study to analyze effects of FOLFOX treatment on non-tumorous human liver tissue at the global protein expression level.

The shotgun proteomics methods used in the study has several strengths and is a useful complement to other global methods in biomedicine [26]. Its global character helps to minimize confirmation bias, similar to microarray techniques. When the complex tissue is analyzed, isolation and preparation of the more stable proteins for LC-MS/MS is advantageous, if compared to limited quality and quantity of isolated RNA for microarray studies. Protein quantification provides insight to the results of gene expression analysis and also reflects posttranscriptional regulation [27], while the amount of mRNA copies does not necessarily reflect the amount of translated protein [28].

Acute hepatocyte injury in cultivated rat hepatocytes after exposure to cisplatin (a platinum-based cytostatic similar to oxaliplatin) for 24 h revealed significant changes in 29% (95/325) of the quantified proteins [25]. In contrast, our data reflect proteome changes 6 weeks after oxaliplatin exposure and only showed changes in about 1% (55/5891) of quantified proteins. Hepatocytes occupy almost 80% and all the other cell populations only 6.5% of total liver volume [29], which implies that observed proteome changes were mostly reflecting changes in hepatocytes. This leads to the hypothesis that recovery of liver tissue from acute FOLFOX injury during the 6 weeks between the last treatment and surgery (time of tissue sampling) minimized the effects on proteome. Similarly, indirect clinical signs of FOLFOX injury, like splenomegaly, reverse after cessation of chemotherapy [9]. In addition, discrepancy between proportions of proteome changes can be partially explained by the variability of the whole liver tissue proteome, which can conceal changes observable in separated cell populations [25].

The major mechanism of oxaliplatin is the formation of platinum–DNA adducts leading to retarded replication and transcription, and ultimately to apoptotic cell death [30]. Mitosis and apoptosis rates in liver is normally low (<0.1%), which makes their estimation difficult [31], but significant changes in DNA replication [32] and MCM complex [33] were observed in the treated group. Increased expression of MCM2 is a sensitive marker of cell cycle entry [34]. The changes identified using stringent statistics (Welch *t*-test) may represent a compensatory effect/recovery of non-tumorous liver tissue from FOLFOX effects [10]. The association of FOLFOX treatment with changes in the expression of proteins involved in the innate immune response is more complex, since identified proteins interacted within the whole network of proteins (Figure 4). The involved proteins, (Table 2), point to processes of oxidative-stress response and ischemia-induced cell death (CAMK2B, VNN1), apoptosis (BCL2, LGALS3), complement activation (C4B, MBL2), and extracellular matrix remodeling (VCAN, NCAM1). These findings are in agreement with previous findings regarding the importance of oxidative stress [16,17,18,19], and extracellular matrix remodeling [21] in the molecular pathway of oxaliplatin. Despite the possibility that changes in nonspecific stress and immune response may lead to subordinated processes, none were verified in enrichment analysis. This suggests that initial signaling is attenuated and does not proceed to further changes in liver proteome, or alternatively, that these changes were not recognized due to interindividual variability and small study groups. Finally, the most probable explanation to the observed cell cycle entry is the hypothesis that only the proteome changes remaining after recovery from the effects of FOLFOX treatment were observed, which agrees with the small proportion of proteome changes noted. Cell cycle entry may also reflect the onset of nodular regenerative hyperplasia, one of the SI histopathological patterns [5].

Identified proteome changes were mostly in the lower part of the LC–MS/MS dynamic range (Figure 2B and Figure 3A), which may be influenced by missing data imputation. Comparison with studies primarily focused on SI-associated transcriptome changes [11,15] is problematic, since no pathological evaluation of SI was performed in the present study. Pathways recognized to be important for SI development in a microarray study by Rubbia-Brandt et al. did not overlap with our proteome analysis. This is probably due to the use of both patients with and without FOLFOX treatment in the control group (without SI) in the microarray analysis [15]. Nevertheless, the classifying proteins showed small, but significant (Fisher exact test *p*-value 0.047) overlap with genes associated with SI in a microarray study by Agostini et al. [11]. The overlap of three proteins, namely COL3A1, VCAN, and TMPRSS6, was significant, despite that only one third (26/81) of the original list of genes was identified in the present study. These three proteins are important in extracellular matrix remodeling, but as mentioned above, no association to such an enrichment category was observed.

## 4. Materials and Methods

### 4.1. Patients and Liver Tissue Samples

Normal liver tissue samples were obtained from patients undergoing a major liver resection for CRLM at Uppsala University Hospital (Uppsala, Sweden) between 2009 and 2012 (Uppsala Regional Ethical approval No. 2009/028). All donors gave their informed consent. The non-tumorous liver tissue samples were cut directly in the operation room after the liver specimen was out of the patient, and immediately stored at −80 °C until further proteomic analysis was conducted.

### 4.2. Preparation of Tissue Lysates

Thawed pieces (about 50 mg) of human liver tissue were homogenized on ice in 0.1 M Tris-HCl, pH 8.0, containing 0.1 M DTT using T10 basic Ultra Turrax blender (IKA, Staufen, Germany) for 10–20 s. The ratio of buffer to tissue was 12:1. To lyse the homogenate, SDS was added to a final concentration of 2% and the mixtures were immediately placed in a boiling water bath (100 °C) for 5 min. After chilling to room temperature, the lysates were clarified by centrifugation at 16,000× *g* for 10 min. Protein concentration in the lysates was determined by the tryptophan fluorescence assay [35]. Each lysate was processed and analyzed in duplicate.

### 4.3. Sample Processing

Aliquots of the liver lysates containing 100 µg total protein were separated from the detergent and DTT using the filter-aided sample preparation (FASP) procedure [36] using 30 k ultrafiltration units (MRCF0R030, Millipore, Merck KGaA, Darmstadt, Germany). Thiol-moieties of proteins were alkylated with 0.05 M iodoacetamide. Cleavage of proteins was carried out by a consecutive two-step digestion with endoproteinase LysC (WAKO Chemicals, Neuss, Germany) and trypsin (Invitrogen, now Thermo Fisher Scientific, Waltham, MA USA) as described previously [37]. The weight ratio of the total protein to the proteinases was 100:1. The yields of the protein to peptide conversion were >75%.

### 4.4. Removal of Substances Affecting Liquid Chromatography–Tandem Mass Spectrometry (LC–MS/MS)

Five microgram aliquots of the LysC and tryptic peptides were brought to pH 11 with 1 × Britton & Robinson Universal Buffer (BRUB) containing 0.1 M CH_3_COOH, 0.1 M H_3_PO_4_, and 0.1 M H_3_BO_3_ adjusted with NaOH to pH 11. The samples were subsequently loaded into pipette-tip-SAX microcolumns as described previously [38]. The columns were washed with 200 µL of 0.2 × BRUB pH 11. The flow-through fractions were discarded. Peptides were eluted with 200 µL of 0.2 × BRUB pH 2 (adjusted with NaOH) and collected in C_18_ –StageTips [39]. Finally, the peptides were released from the Stage Tips with 40 µL 60% CH_3_CN in water. The organic solvent was evaporated and the volumes of the peptide solutions were reduced to about 5 µL in a speed-vac. This step was essential for reproducible liquid chromatography and efficient MS^2^ –peptide identification (LC-MS/MS).

### 4.5. LC–MS/MS and Raw Data Analysis

Peptides were separated on a reverse-phase column (20 cm × 75 μm inner diameter) packed in-house with 1.8 μm C18 particles (Maisch, Ammerbuch-Entringen, Germany) using a 4 h ACN gradient in 0.1% formic acid at a flow rate of 250 nL/min. The column was operated at a constant temperature of 35 °C. The LC was coupled to a Q Exactive mass spectrometer (Thermo Fisher Scientific, Waltham, MA, USA) via the nanoelectrospray source (Thermo Fisher Scientific). The Q Exactive was operated in the data-dependent mode with survey scans acquired at a resolution of 50,000 at *m*/*z* 400 (transient time 256 ms). Up to the top 10 most abundant isotope patterns with charge ≥2 from the survey scan were selected with an isolation window of 1.6 Th and fragmented by higher energy collisional dissociation with normalized collision energies of 25. The maximum ion injection times for the survey scan and the MS/MS scans were 20 and 60 ms, respectively, and the ion target value for both scan modes were set to 10^6^. The spectra were analyzed using the Max Quant software (version 1.2.2.8, Max-Planck-Institute of Biochemistry, Martinsried, Germany; http://www.coxdocs.org) with the “matching between runs” option. The proteins were identified by searching MS and MS/MS data of peptides against UniProtKB human database (version April 2013; http://www.uniprot.org). Carboamidomethylation of cysteines was set as fixed modification. The minimum peptide length was specified to be seven amino acids. The initial maximal mass tolerance in MS mode was set to 7 ppm, whereas fragment mass tolerance was set to 20 ppm for fragmentation data. The maximum false peptide discovery rate was specified as 0.01. The FDR threshold was derived by analyzing the decoy database. Max Quant label-free quantification was used.

For proteins that were detected in more than 50% of samples, zero intensities were filled with intensities from the lower part of normal distribution (imputation width = 0.3, shift = 1.8) using Perseus 1.4.1.3 software (Max-Planck-Institute of Biochemistry, Martinsried, Germany; http://www.coxdocs.org).

### 4.6. Statistical Analysis

Clinical data were described using proportions, median, and interquartile range (IQR). Differences between groups were judged by Fisher exact test or Mann–Whitney test as appropriate in IBM^®^SPSS^®^Statistics 22.0 (Armonk, NY, USA). Proteome data analysis was performed using Perseus software. To assess intra- and interindividual proteome variability, unsupervised hierarchical clustering (on z-scored data) according to average Euclidean distance and principal component analysis (PCA) were performed for all the technical replicates. Classification feature optimization was used to identify classifying proteins between the study groups. All the proteins were ranked according their classification ability using recursive feature elimination-support vector machine (RFE-SVM) (standard parameters c = 100, reduction factor 1.414) and cross-validated according to the leave-one-out method by RFE–SVM (linear Kernel, c = 10, size reduction factor = 1.414). The best classifying proteins were identified as the smallest set of highly ranked proteins achieving minimal classification error rate. Differently expressed proteins were identified using Welch *t*-test corrected for false discovery ratio (FDR) < 5% counted by permutation method with 5000 runs and manually tuned exchangeability factor s_0_. The method prevented influence of possible technical bias by strictly omitting technical replicates from permutations counting [40]. Optimal s_0_ (percentile of the standard deviation values of all the proteins) ensures that both significance and fold change is taken into account through adjustment of the significance threshold. In brief, the significance level is lowered for proteins displaying a high fold change and raised for proteins with a small fold change. The lists of classifying and relevantly changed proteins were analyzed for physical and functional associations using STRING 9.1 (String Consortium; http://string-db.org). Connections were expressed as a sum of functional evidence and action scores. Overlaps with gene ontology categories and pathways were expressed using FDR-adjusted *p*-value. Adjusted or unadjusted (when appropriate) *p*-values of less than 0.05 were recognized as significant.

## 5. Conclusions

Only a small proportion (~1%) of the proteome of non-tumorous human liver parenchyma was changed in patients 6 weeks after FOLFOX treatment. Changes were associated with cell cycle entry through MCM complex activity and with the innate immune response. This indicates that these are the only remaining proteome changes that persist in liver parenchyma after recovery from the FOLFOX treatment.

## Figures and Tables

**Table 1:** List of relevantly changed proteins according to Welch *t*-test, at false discovery ratio < 0.05, s_0_ = 0.05. Numbers of identified peptides and unique peptides are also provided.

Gene Names	Protein Names	Welch *t*-Test *p*-Value	Fold Change	Coefficient of Variation	Peptides	Unique Peptides
MAP1B	Microtubule-associated protein 1B;MAP1 light chain LC1	0.007	10.21	1.43	24	23
HLA-DQA1	Major histocompatibility complex, class II, DQ alpha 1	0.002	6.23	1.12	4	2
C19orf52	Uncharacterized protein C19orf52	0.012	4.90	1.92	3	3
IGHD	Ig delta chain C region	0.022	3.88	1.82	7	7
MCM2	DNA replication licensing factor MCM2	0.002	3.40	0.80	12	12
MLIP	Muscular LMNA-interacting protein	0.001	3.11	0.74	4	4
STMN2	Stathmin-2	0.021	3.02	0.93	1	1
Q7Z7K6	Centromere protein V	0.013	2.84	1.37	8	3
MCM4	DNA replication licensing factor MCM4	0.007	2.71	0.82	7	7
EMG1	Ribosomal RNA small subunit methyltransferase NEP1	0.017	2.63	1.17	4	4
NUDT12	Peroxisomal NADH pyrophosphatase NUDT12	0.009	2.38	0.73	8	8
DHFR; DHFRL1	Dihydrofolate reductase;Dihydrofolate reductase, mitochondrial	0.014	2.31	0.62	2	2
OSBPL6	Oxysterol-binding protein-related protein 6	0.010	2.25	0.84	3	3
MCM7	DNA replication licensing factor MCM7	0.012	2.20	0.71	11	11
ANGPTL3	Angiopoietin-related protein 3	0.009	2.14	0.62	5	5
TMEM2	Transmembrane protein 2	0.002	2.02	0.51	4	4
DDX20	Probable ATP-dependent RNA helicase DDX20	0.006	1.94	0.54	4	4
ISG15	Ubiquitin-like protein ISG15	0.010	1.82	0.67	5	5
CCDC25	Coiled-coil domain-containing protein 25	0.004	1.79	0.59	9	9
NBEAL1	Neurobeachin-like protein 1	0.009	1.63	0.52	10	10
BCO2	Beta,beta-carotene 9,10-oxygenase	0.009	1.57	0.43	22	22
HAL	Histidine ammonia-lyase	0.013	−1.65	0.46	25	25
ASAH1	Acid ceramidase;Acid ceramidase subunit alpha;Acid ceramidase subunit beta	0.000	−1.66	0.38	15	15
CYP2S1	Cytochrome P450 2S1	0.008	−1.72	0.45	2	2
GPX1	Glutathione peroxidase 1	0.011	−1.76	0.55	13	13
CLEC16A	Protein CLEC16A	0.013	−1.76	0.60	1	1
ACTR1B	Beta-centractin	0.015	−1.83	0.51	9	4
CHMP1A	Charged multivesicular body protein 1a	0.009	−1.87	0.56	1	1
CTBS	Di-*N*-acetylchitobiase	0.012	−1.98	0.71	5	5
FOLH1; FOLH1B	Glutamate carboxypeptidase 2;Putative *N*-acetylated-alpha-linked acidic dipeptidase	0.014	−2.01	0.74	8	8
KHNYN	Protein KHNYN	0.002	−2.02	0.59	2	2
FRG1	Protein FRG1	0.017	−2.17	0.92	3	3
SRP72	Signal recognition particle 72 kDa protein	0.001	−2.22	0.68	19	19
PLSCR3	Phospholipid scramblase 3	0.012	−2.23	0.56	2	2
ERF	ETS domain-containing transcription factor ERF	0.013	−2.25	0.66	3	3
FNBP1	Formin-binding protein 1	0.014	−2.28	0.75	5	5
SEPP1	Selenoprotein P	0.018	−2.34	0.69	2	2
RHPN2	Rhophilin-2	0.009	−2.40	0.89	4	4
ELOVL1	Elongation of very long chain fatty acids protein 1	0.019	−2.46	1.61	2	2
MRPS7	28S ribosomal protein S7, mitochondrial	0.015	−2.48	0.76	9	9
RIN1	Ras and Rab interactor 1	0.012	−2.82	0.55	2	2
ITIH5	Inter-alpha-trypsin inhibitor heavy chain H5	0.008	−2.95	1.23	3	3
CAMK2G; CAMK2A; CAMK2B	Calcium/calmodulin-dependent protein kinase type II subunit gamma;Calcium/calmodulin-dependent protein kinase type II subunit alpha	0.011	−2.96	0.88	8	2
NT5DC2	5-nucleotidase domain-containing protein 2	0.006	−3.04	0.92	4	4
FAN1	Fanconi-associated nuclease 1	0.011	−3.09	1.02	3	3
OXNAD1	Oxidoreductase NAD-binding domain-containing protein 1	0.003	−3.20	1.04	5	5
ALOX5AP	Arachidonate 5-lipoxygenase-activating protein	0.014	−3.34	1.25	2	2
MACF1	Microtubule-actin cross-linking factor 1, isoforms 1/2/3/5	0.016	−3.52	1.57	83	0
CPA3	Mast cell carboxypeptidase A	0.008	−3.67	1.72	4	4
KRT80	Keratin, type II cytoskeletal 80	0.005	−3.67	0.64	5	4
MYBPC2	Myosin-binding protein C, fast-type	0.004	−4.29	0.96	4	3
ERAP2	Endoplasmic reticulum aminopeptidase 2	0.022	−4.59	0.99	20	20
YOD1	Ubiquitin thioesterase OTU1	0.017	−4.95	1.56	3	3
TLCD1	TLC domain-containing protein 1	0.022	−5.37	2.22	2	2
UBE3B	Ubiquitin-protein ligase E3B	0.024	−6.17	0.96	7	7

**Table 2:** Gene ontology and pathways associated with significantly changed or classifying proteins.

Category	Term	Welch *t*-Test Significant Proteins (*n* = 55)			Classifying Model Selected Proteins (*n* = 184)		
		*p*-Value	*p*-Value FDR	Intersection Genes	*p*-Value	*p*-Value FDR	Intersection Genes
Biological Process	DNA unwinding involved in replication	<0.001	0.013	MCM2; MCM4; MCM7	<0.001	0.007	MCM2; MCM4; MCM6; MCM7
	innate immune response				<0.001	0.029	BCL2; C4B; CAMK2B; CD4; ENSG00000228284; HCK; HLA-DR4; IGKV4-1; IGLV7-43; ISG15; LGALS3; MAPKAPK3; MBL2; MX1; NCAM1; RPS6KA1; VNN1
Cellular Component	MCM complex				<0.001	<0.001	MCM4; MCM5; MCM6; MCM7
Pathway	DNA replication	<0.001	0.013	MCM2; MCM4; MCM7	<0.001	0.021	MCM2; MCM4; MCM5; MCM6; MCM7

## Supplementary Materials

The following are available online at www.mdpi.com/2227-7382/4/4/30/s1, Table S1: Clinical data, Table S2: Classifying proteins according Recursive Feature Elimination-Support Vector Machine model resulting in list of 184 proteins with classifying error rate 20%. RAW files and the MaxQuant search results were deposited in PRIDE repository database [41] with the dataset identifier: PXD001889.

## Abbreviations

The following abbreviations are used in this manuscript:

CRLM: Colo-Rectal cancer Liver Metastases
FOLFOX: Fluorouracil Leucovorin Oxaliplatin treatment
SI: Sinusoidal Injury
PCA: Principal Component Analysis
RFE-SVM: Recursive Feature Elimination-Support Vector Machine
FDR: False Discovery Ratio
MCM: minichromosome maintenance complex
LC-MS/MS: Liquid chromatography-tandem mass spectrometry
