# Supplementary material for: Global Proteome Changes in Liver Tissue 6 Weeks after FOLFOX Treatment of Colorectal Cancer Liver Metastases
Source: Proteomes. 2016 Oct 14;4(4):30. doi: 10.3390/proteomes4040030 (PMC5260963; doi:10.3390/proteomes4040030)
Supplement: Supplementary file 1 [file proteomes-04-00030-s001.pdf]

Supplementary Materials: Global Proteome Changes in Liver Tissue 6 Weeks After FOLFOX Treatment of Colorectal Cancer Liver Metastases

Jozef Urdzik, Anna Vildhede, Jacek R. Wiśniewski, Frans Duraj, Ulf Haglund, Per Artursson and Agneta Norén

Table S1. Clinical data.

Clinical Characteristics	Expressed By	All Patients (*n* = 15)	FOLFOX Treatment (*n* = 8)	No Chemotherapy (*n* = 7)	*p*-Value
Gender (male)	n (%)	11 (73%)	5 (63%)	6 (86%)	0.57
Age (years)	median, IQR	59 (58–69)	63 (58–70)	58 (53–70)	0.40
BMI	median, IQR	25.6 (24.1–30.0)	25.0 (23.7–27.6)	29.8 (24.2–31.0)	0.19
Number of FOLFOX cures	median, IQR	-	5 (4–7)	-	
Delay after FOLFOX cessation (weeks)	median, IQR	-	6 (5–8)	-	
Major liver resection	n (%)	12 (80%)	7 (88%)	5 (71%)	0.57
Bleeding preoperatively (mL)	median, IQR	600 (300–1700)	450 (300–600)	1100 (400–2200)	0.12
Transfusion units peroperatively	median, IQR	0 (0–0)	0 (0–0)	0 (0–0)	1.00
Transfusion units postoperatively	median, IQR	0 (0–0)	0 (0–0)	0 (0–0)	0.69
Hospital stay (days)	median, IQR	10 (8–15)	10 (8–14)	10 (7–16)	0.78
Clavien complication grade 3 or more	n (%)	5 (33%)	2 (25%)	3 (43%)	0.61

Table S2. Classifying proteins according Recursive Feature Elimination-Support Vector Machine model resulting in list of 184 proteins with classifying error rate 20%.

Gene Names Alt. Protein ID	Protein Names	Ranks	Fold Change	Peptides	Unique Peptides
MCM2	DNA replication licensing factor MCM2	0	3.40	12	12
CAMK2G; CAMK2A; CAMK2B	Calcium/calmodulin-dependent protein kinase type II subunit gamma; Calcium/calmodulin-dependent protein kinase type II subunit alpha	1	−2.96	8	2
VCAN	Versican core protein	2	−2.86	10	10
FBXO3	F-box only protein 3	3	3.58	8	8
MYH15	Myosin-15	4	−2.28	3	2
UGT2B17; F8WCE9	UDP-glucuronosyltransferase 2B17; UDP-glucuronosyltransferase 2B15	5	5.40	25	1
OXNAD1	Oxidoreductase NAD-binding domain-containing protein 1	6	−3.20	5	5
CXorf27	Huntingtin-interacting protein M	7	3.94	1	1
MAP1B	Microtubule-associated protein 1B; MAP1 light chain LC1	8	10.21	24	23
C4B	Complement component 4B	9	−2.74	67	0
TMPRSS6	Transmembrane protease serine 6	10	6.60	4	4
TLCD1	TLC domain-containing protein 1	11	−5.37	2	2
MB	Myoglobin	12	2.30	4	4
UBE3B	Ubiquitin-protein ligase E3B	13	−6.17	7	7
MYBPC2	Myosin-binding protein C, fast-type	14	−4.29	4	3
GSTT2; GSTT2B; Em:AP000351.3	Glutathione S-transferase theta-2; Glutathione S-transferase theta-2B	15	−4.27	7	6
OLFML1	Olfactomedin-like protein 1	16	2.89	13	13
CPA3	Mast cell carboxypeptidase A	17	−3.67	4	4

Table S2. *cont.*

Gene Names Alt. Protein ID	Protein Names	Ranks	Fold Change	Peptides	Unique Peptides
MACF1	Microtubule-actin cross-linking factor 1, isoforms 1/2/3/5	18	−3.52	83	0
MX1	Interferon-induced GTP-binding protein Mx1; Interferon-induced GTP-binding protein Mx1, *N*-terminally processed	19	1.88	16	14
BCL2	Apoptosis regulator Bcl-2	20	−2.34	3	3
SASH1	SAM and SH3 domain-containing protein 1	21	−3.41	2	2
ERAP2	Endoplasmic reticulum aminopeptidase 2	22	−4.59	20	20
C19orf12	Chromosome 19 open reading frame 12	23	−2.17	4	2
CMA1	Chymase	24	−3.35	4	4
ALOX5AP	Arachidonate 5-lipoxygenase-activating protein	25	−3.34	2	2
TRAPPC5	Trafficking protein particle complex subunit 5	26	−1.57	3	3
CD4	T-cell surface glycoprotein CD4	27	2.26	2	2
SMYD5	SET and MYND domain-containing protein 5	28	4.62	3	3
ICT1	Peptidyl-tRNA hydrolase ICT1, mitochondrial	29	−3.36	4	4
MLIP; C6orf142	Muscular LMNA-interacting protein	30	3.11	4	4
TMCO4	Transmembrane and coiled-coil domain-containing protein 4	31	1.70	2	2
DUOX2	Dual oxidase 2	32	−2.59	2	2
RHPN2	Rhophilin-2	33	−2.40	4	4
TM9SF4	Transmembrane 9 superfamily member 4	34	2.47	6	6
YOD1	Ubiquitin thioesterase OTU1	35	−4.95	3	3
FAN1	Fanconi-associated nuclease 1	36	−3.09	3	3
KIAA1586	Uncharacterized protein KIAA1586	37	−2.30	1	1
HLA-DQA1	Major histocompatibility complex, class II, DQ alpha 1	38	6.23	4	2
NAT2	Arylamine *N*-acetyltransferase 2	39	−2.67	6	6
GYS1	Glycogen [starch] synthase, muscle	40	−2.30	12	9
XRN1	5-3 exoribonuclease 1	41	−2.72	7	7
ALG13	UDP-*N*-acetylglucosamine transferase subunit ALG13 homolog	42	−2.66	2	2
SEPP1	Selenoprotein P	43	−2.34	2	2
HYI	Putative hydroxypyruvate isomerase	44	2.32	6	5
PLEKHA4	Pleckstrin homology domain-containing family A member 4	45	2.15	10	10
RAB5A; RAB5B	Ras-related protein Rab-5A	46	−2.30	6	3
KRT80	Keratin, type II cytoskeletal 80	47	−3.67	5	4
MYOM2	Myomesin-2	48	1.55	13	13
PODN	Podocan	49	2.75	5	5
BCHE	Cholinesterase	50	−2.07	3	3
FSIP2	Fibrous sheath-interacting protein 2	51	3.61	7	7
PRG2	Bone marrow proteoglycan; Eosinophil granule major basic protein	52	3.42	6	6
CLDN5	Claudin-5	53	−2.04	1	1
CIT	Citron Rho-interacting kinase	54	−2.95	7	7
UTP20	Small subunit processome component 20 homolog	55	−2.42	6	6
MCM4	DNA replication licensing factor MCM4	56	2.71	7	7
PLSCR3	Phospholipid scramblase 3	57	−2.23	2	2
Q7Z7K6-3; Q7Z7K6-2	Centromere protein V	58	2.84	8	3
TMEM2	Transmembrane protein 2	59	2.02	4	4
PMM2	Phosphomannomutase 2	60	−2.23	5	1
ARSE	Arylsulfatase E	61	2.41	9	8
NT5DC2	5-nucleotidase domain-containing protein 2	62	−3.04	4	4
NLRP13	NACHT, LRR and PYD domains-containing protein 13	63	−4.24	3	3
GSTT1	Glutathione S-transferase theta-1	64	2.50	15	11
PRPSAP1	Phosphoribosyl pyrophosphate synthase-associated protein 1	65	2.19	14	1
ENAH	Protein enabled homolog	66	−1.67	7	7
DNAH3	Dynein heavy chain 3, axonemal	67	2.32	5	5

Table S2. *cont.*

Gene Names Alt. Protein ID	Protein Names	Ranks	Fold Change	Peptides	Unique Peptides
ALS2CL	ALS2 *C*-terminal-like protein	68	1.94	3	3
HLA-F	HLA class I histocompatibility antigen, alpha chain F	69	1.68	6	5
INTS4	Integrator complex subunit 4	70	−2.40	5	5
IGHD	Ig delta chain C region	71	3.88	7	7
UAP1L1	UDP-*N*-acetylhexosamine pyrophosphorylase-like protein 1	72	1.66	7	7
TKT	Transketolase	73	−2.73	29	1
PLIN1	Perilipin-1	74	2.06	14	14
STX7	Syntaxin-7	75	1.80	5	5
GSTM1	Glutathione S-transferase Mu 1	76	3.52	21	7
EXOC3	Exocyst complex component 3	77	−2.15	4	4
ARHGEF18	Rho guanine nucleotide exchange factor 18	78	−2.07	4	4
FOLH1; FOLH1B	Glutamate carboxypeptidase 2; Putative *N*-acetylated-alpha-linked acidic dipeptidase	79	−2.01	8	8
NCF1B; NCF1; NCF1C	Putative neutrophil cytosol factor 1B; Neutrophil cytosol factor 1; Putative neutrophil cytosol factor 1C	80	−3.39	8	8
LGALS3	Galectin-3	81	−1.99	6	6
MCM7	DNA replication licensing factor MCM7	82	2.20	11	11
SET	Protein SET	83	−2.26	7	1
NDUFS8	NADH dehydrogenase (ubiquinone) Fe-S protein 8, 23kDa (NADH-coenzyme Q reductase)	84	2.27	5	1
TBC1D23	TBC1 domain family member 23	85	2.37	4	4
MYL10	Myosin regulatory light chain 10	86	2.08	1	1
DHFR; DHFRL1	Dihydrofolate reductase; Dihydrofolate reductase, mitochondrial	87	2.31	2	2
RPL26L1	60S ribosomal protein L26-like 1	88	1.99	9	2
ITIH5	Inter-alpha-trypsin inhibitor heavy chain H5	89	−2.95	3	3
TFPI	Tissue factor pathway inhibitor	90	−2.21	3	3
HADHB	Hydroxyacyl-CoA dehydrogenase/3-ketoacyl-CoA thiolase/enoyl-CoA hydratase (trifunctional protein), beta subunit	91	−1.69	3	1
KIAA1967	KIAA1967-potential transmembrane protein	92	1.82	5	1
TMEM176B	Transmembrane protein 176B	93	1.95	2	2
COL3A1	Collagen alpha-1(III) chain	94	−1.87	6	6
APOL3	Apolipoprotein L3	95	1.96	5	5
ANXA6	Annexin A6; Annexin	96	2.20	55	1
MAGI1	Membrane-associated guanylate kinase, WW and PDZ domain-containing protein 1	97	−1.98	3	3
TRIOBP	TRIO and F-actin-binding protein	98	−1.74	7	2
NCAM1	Neural cell adhesion molecule 1	99	−2.12	8	8
ANGPTL6	Angiopoietin-related protein 6	100	2.02	9	9
RIN1	Ras and Rab interactor 1	101	−2.82	2	2
CUL4B	Cullin-4B	102	2.13	15	9
ERBB2	Receptor tyrosine-protein kinase erbB-2	103	1.83	7	7
SPTAN1	Spectrin, alpha, non-erythrocytic 1	104	2.59	143	1
ATP2A3	Sarcoplasmic/endoplasmic reticulum calcium ATPase 3	105	−2.27	17	8
MRPS7	28S ribosomal protein S7, mitochondrial	106	−2.48	9	9
THOC1	THO complex subunit 1	107	−2.00	6	6
ZNF326	Zinc finger protein 326	108	1.84	7	7
JMJD7	JmjC domain-containing protein 7	109	1.61	3	3
ESAM	Endothelial cell-selective adhesion molecule	110	−1.57	4	4
UBE2H	Ubiquitin-conjugating enzyme E2 H	111	−1.51	3	3
EMG1	Ribosomal RNA small subunit methyltransferase NEP1	112	2.63	4	4
VRK2	Serine/threonine-protein kinase VRK2	113	2.22	4	4
C1GALT1C1	C1GALT1-specific chaperone 1	114	−2.18	2	2
P06314; P01625; P83593	Ig kappa chain V-IV region B17; Ig kappa chain V-IV region Len	115	−2.24	3	1
ARHGEF11	Rho guanine nucleotide exchange factor 11	116	2.67	3	3
SUSD2	Sushi domain-containing protein 2	117	1.56	4	4

Table S2. *cont.*

Gene Names Alt. Protein ID	Protein Names	Ranks	Fold Change	Peptides	Unique Peptides
C19orf52	Uncharacterized protein C19orf52	118	4.90	3	3
GGA2	ADP-ribosylation factor-binding protein GGA2	119	−2.10	6	4
NUDT12	Peroxisomal NADH pyrophosphatase NUDT12	120	2.38	8	8
SRD5A2	3-oxo-5-alpha-steroid 4-dehydrogenase 2	121	−1.77	6	6
TGM1	Protein-glutamine gamma-glutamyltransferase K	122	−1.78	5	5
ELOVL1	Elongation of very long chain fatty acids protein 1	123	−2.46	2	2
GALNT1	Polypeptide *N*-acetylgalactosaminyltransferase 1; Polypeptide *N*-acetylgalactosaminyltransferase 1 soluble form	124	−1.68	4	4
HLA-DRB1	HLA class II histocompatibility antigen, DRB1-10 beta chain	125	−2.03	4	1
GOLPH3	Golgi phosphoprotein 3	126	−2.22	6	6
P23083	Ig heavy chain V-I region V35	127	−2.03	2	2
TMF1	TATA element modulatory factor	128	−2.45	3	3
NAA38	*N*-alpha-acetyltransferase 38, NatC auxiliary subunit	129	−2.18	3	3
NXN	Nucleoredoxin	130	−1.71	3	3
CA14	Carbonic anhydrase 14	131	2.06	4	4
ERF	ETS domain-containing transcription factor ERF	132	−2.25	3	3
SLC39A7	Zinc transporter SLC39A7	133	2.56	2	2
PRPF38A	Pre-mRNA-splicing factor 38A	134	2.25	4	4
EXOSC5	Exosome complex component RRP46	135	1.72	2	2
GCKR	Glucokinase regulatory protein	136	−2.27	18	18
STMN2; STMN1	Stathmin; Stathmin-2	137	3.02	1	1
CTIF	CBP80/20-dependent translation initiation factor	138	−1.59	3	3
PCDHB12	Protocadherin beta-12	139	−2.26	1	1
EPG5	Ectopic P granules protein 5 homolog	140	1.69	5	5
DOCK5	Dedicator of cytokinesis protein 5	141	−1.89	7	7
SRBD1	S1 RNA-binding domain-containing protein 1	142	−2.01	6	6
DDX3Y	ATP-dependent RNA helicase DDX3Y	143	−2.11	16	1
CYP2D6	Cytochrome P450 2D6	144	2.71	24	24
FRG1	Protein FRG1	145	−2.17	3	3
PWP2	Periodic tryptophan protein 2 homolog	146	−1.61	7	7
RPS6KA1	Ribosomal protein S6 kinase alpha-1; Ribosomal protein S6 kinase	147	−2.61	11	5
LGALS8	Galectin-8	148	1.62	5	5
MBL2	Mannose-binding protein C	149	−2.14	7	7
ACOT9	Acyl-coenzyme A thioesterase 9, mitochondrial	150	−2.11	13	13
TTC17	Tetratricopeptide repeat protein 17	151	−1.81	4	4
C20orf7	Probable methyltransferase C20orf7, mitochondrial	152	−2.34	4	4
P04211	Ig lambda chain V region 4A	153	−1.97	1	1
LMOD1	Leiomodin-1	154	−2.45	6	6
MT-ND6	NADH-ubiquinone oxidoreductase chain 6	155	1.76	1	1
LAMA4	Laminin subunit alpha-4	156	−2.04	16	16
HLA-DRB1	HLA class II histocompatibility antigen, DRB1-4 beta chain	157	2.19	9	1
FBXO2	F-box only protein 2	158	2.23	4	4
RPS4Y1	40S ribosomal protein S4, Y isoform 1	159	−1.60	16	4
ISG15	Ubiquitin-like protein ISG15	160	1.82	5	5
POP7	Ribonuclease P protein subunit p20	161	1.82	1	1
ANKS1A	Ankyrin repeat and SAM domain-containing protein 1A	162	−2.57	3	3
MCM3AP	80 kDa MCM3-associated protein	163	3.40	3	3
KCMF1	E3 ubiquitin-protein ligase KCMF1	164	2.25	1	1
MECP2	Methyl-CpG-binding protein 2	165	−1.91	7	7
TRMT11	tRNA (guanine(10)-*N*2)-methyltransferase homolog	166	−1.69	4	4
MAPKAPK3	MAP kinase-activated protein kinase 3	167	−1.49	5	4
PTPRM	Receptor-type tyrosine-protein phosphatase mu	168	−1.87	4	2
MCM6	DNA replication licensing factor MCM6	169	1.80	6	6
DDX28	Probable ATP-dependent RNA helicase DDX28	170	−1.42	3	3
VNN1	Pantetheinase	171	1.86	6	6

Table S2. *cont.*

Gene Names Alt. Protein ID	Protein Names	Ranks	Fold Change	Peptides	Unique Peptides
ACTB	Actin, beta	172	−2.07	14	2
MADD	MAP kinase-activating death domain protein	173	−2.17	4	4
HNRPLL	Heterogeneous nuclear ribonucleoprotein L-like	174	−1.95	5	5
ANGPTL3	Angiopoietin-related protein 3	175	2.14	5	5
SCRN1	Secernin-1	176	2.01	4	4
TOR2A	Torsin-2A	177	1.63	6	5
MCM5	DNA replication licensing factor MCM5	178	1.94	9	9
ALDH3B1	Aldehyde dehydrogenase family 3 member B1	179	−1.93	5	4
HCK	Tyrosine-protein kinase HCK	180	−1.74	10	5
KHNYN	Protein KHNYN	181	−2.02	2	2
F10	Coagulation factor X; Factor X light chain; Factor X heavy chain; Activated factor Xa heavy chain	182	1.79	6	6
TRAPPC10	Trafficking protein particle complex subunit 10	183	−1.49	5	5
